# Cyanobacterial Catalase Activity Prevents Oxidative Stress Induced by *Pseudomonas fluorescens* DUS1-27 from Inhibiting *Brassica napus* L. (canola) Growth

**DOI:** 10.1264/jsme2.ME18061

**Published:** 2018-11-23

**Authors:** Lee Hudek, Aydin Enez, Lambert Bräu

**Affiliations:** 1 Deakin University, Geelong Australia Centre for Regional and Rural Futures, School of Life and Environmental Sciences; 2 Deakin University, Geelong Australia Centre for Cellular and Molecular Biology, School of Life and Environmental Sciences

**Keywords:** reactive oxygen species (ROS), *Brassica napus* L. (canola), *Pseudomonas fluorescens*, *Nostoc punctiforme*

## Abstract

Plant growth-promoting bacteria (PGPB) inhabit the rhizosphere of plants and are capable of enhancing plant growth through a number of mechanisms. A strain of *Pseudomonas fluorescens* DUS1-27 was identified as a potential PGPB candidate based on its ability to increase the growth of *Brassica napus* L. (canola) over that of uninoculated control plants in a soil-based system. The same *P. fluorescens* isolate was found to reduce plant growth in a hydroponic growth system, with plants showing the symptoms of a microbe-associated molecular pattern (MAMP) response to the bacteria. The amperometric quantification of H_2_O_2_, fluorescence-based total peroxidase assays, and quantification of catalase gene expression levels using qRT-PCR revealed that oxidative stress reduced plant growth in the hydroponic system. The addition of the cyanobacterium *Nostoc punctiforme* (known to have high catalase activity levels) in the hydroponic system as a co-inoculant reduced oxidative stress (49.7% decrease in H_2_O_2_ concentrations) triggered by the addition of *P. fluorescens* DUS1-27, thereby enabling plants to grow larger than uninoculated control plants. These results show the advantage of inoculating with multiple bacteria to promote plant growth and, for the first time, demonstrate that *N. punctiforme* beneficially assists plants under oxidative stress through its catalase activity *in planta*.

Plant growth-promoting bacteria (PGPB) inhabit the rhizosphere of plants and are capable of enhancing plant growth by increasing nutrient availability to plants through their production of siderophores and other peptides, their production of plant growth hormones, such as indole-3-acetic acid (IAA—cell elongation and cell growth factor), and enzymatic activities, including aminocyclicpropane-1-carboxylase (ACC) deaminase (reduction of ethylene-induced stress signaling) and nitrogen fixation (19, 24, 37). While many rhizosphere organisms have been shown to produce the plant growth benefits described above, some may also exert deleterious effects if delivered to the rhizosphere in sufficient numbers (1, 19, 37, 39). High rhizosphere bacterial numbers were previously reported to stunt root development, including root hair formation, leading to overall reductions in plant development or even death (2, 5, 8, 11). This has been attributed to changes in the dynamics of plant-microbe interactions rather than bacteria changing metabolism *per se* (2, 5, 8, 11).

Preliminary investigations identified *P. fluorescens* DUS1-27 as a potential PGPB candidate for *B. napus* based on its ability to solubilize iron and phosphate, produce IAA and ACC deaminase, and significantly increase plant growth over that of an uninoculated control in soil-based medium for a growth period of one month. The inoculation of *B. napus* with *P. fluorescens* DUS1-27 significantly increased dry root and shoot biomasses by approximately 1.7-fold (120 mg) and 1.4-fold (170 mg), respectively, compared to uninoculated control plants.

A hydroponic growth system was used to further characterize the plant growth-promoting (PGP) mechanism(s) of the *P. fluorescens* DUS1-27 strain. Hydroponic growth environments offer greater experimental control by reducing the variability frequently associated with soil-based plant growth media, and also allow a greater range of biochemical analyses to be performed (50, 52, 53). When *B. napus* plants were inoculated with the *P. fluorescens* DUS1-27 isolate and grown in a hydroponic system their growth was significantly reduced. Based on the phenotype of these plants, oxidative stress associated with increased levels of reactive oxygen species (ROS) was identified as the likely candidate for plant growth reductions. ROS may be detrimental to plants, reducing their viability and overall growth via oxidative damage to cellular components, such as lipids, DNA, and proteins (15, 17, 45).

The bacterial inoculation of plants was previously reported to complement their antioxidant activities when exposed to ROS inducing abiotic stressors (28). The high catalase activity levels of *Pseudomonas mendocina* Palleroni have been shown to mitigate the effects of ROS-inducing stressors in *Lactuca sativa* L. (28), suggesting that microbial catalase activity alleviates oxidative stress in other plant systems. Based on the known high catalase capacity of *N. punctiforme* to reduce ROS (27, 33), we investigated whether the co-inoculation of this cyanobacterium in the hydroponic growth system reduces plant growth-limiting oxidative stress induced by *P. fluorescens* DUS1-27 *in planta*.

*Nostoc punctiforme* PCC73120 represents a highly adapted genus, capable of growing in diverse ecosystems from freshwater through to terrestrial locations, and is a multicellular species that produces the following cell types: photosynthetic vegetative cells that form filaments, motile hormogonia (short motile filaments capable of forming symbiotic relationships with plants), heterocysts (nitrogen-fixing cells that facilitate diazotrophic growth), and akinetes (robust spore-like cells). (3, 13). *N. punctiforme* PCC73120 is free living and also forms symbiotic relationships with a broad range of plant hosts, with its nutrient-scavenging abilities fulfilling its required nutrient quota for growth (3, 13).

Previous studies investigated the extensive and functionally diverse enzymes produced by *N. punctiforme* that break down ROS under different stress conditions, including a putative MnCat (re-annotated as putative cytochrome C) (*Npun_F4992*, Acc. No. WP_012411291), the differential expression of superoxide dismutases (SODs) in response to methyl viologen-induced oxidative stress, the characterization of the neighboring heme dioxygenase/peroxidase “Np-diox” (*Npun_R5469*, Acc. No. YP_001868719), the catalase “Np-cat” (*Npun_R5468*, Acc. No. YP_001868718), and the characterization of peroxide reduction by the DNA-binding protein (Dps) ferroxidase (*Npun_F3730*, Acc. No. YP_001867063) and a metal-dependent catalase (*Npun_R4582*, YP_001867885) (12, 16, 27, 33, 40). The capacity for *N. punctiforme* to breakdown ROS rapidly may not only aid in photosynthesis, but also enable it to establish symbiotic relationships with plant hosts or reduce ROS levels in the surrounding environment.

We investigated whether reductions in *B. napus* growth are due to increased ROS levels accumulating in the hydroponic growth system when it is inoculated with *P. fluorescens* DUS1-27, and if a co-inoculation with the high catalase/peroxidase-producing cyanobacterial species, *N. punctiforme*, decreases the plant-growth inhibiting levels of hydrogen peroxide (H_2_O_2_) in both the environment (hydroponic media) and in *B. napus*. The development of co-inoculants for use as biofertilizers may enable the complementation of each PGPB’s ability to enhance plant growth, as well as offer a safeguard against bacterial inoculants having undesirable side effects in agroecosystems.

## Materials and Methods

### *P. fluorescens* DUS1-27 strain and culture conditions

*P. fluorescens* DUS1-27 is a Gram-negative rod-shaped bacterium that was isolated from the rhizosphere of a *B. napus* plant in Victoria, Australia. *P. fluorescens* DUS1-27 cells were cultured in liquid tryptone-yeast extract (TY) medium at 28°C in a shaking incubator (180 rpm) for 24 h or streak plated on TY agar (1.5%) and grown at 28°C for 24 h. Cell stocks were stored frozen at −80°C in TY with 20% added glycerol. Cell numbers were standardized for all experiments by measuring optical density at an absorbance of 600 nm (OD_600_) and confirmed by Miles and Misra plate counts unless otherwise specified.

### *N. punctiforme* PCC73120 strains and culture conditions

Wild-type (WT) *N. punctiforme* PCC73120, previously generated *N. punctiforme* catalase *Npun_R4582^−^* knockout mutant (27), and *N. punctiforme* catalase-overexpressing (*Npun_R4582* OE) mutant (27) stock cultures were grown in 500 mL of BG11 broth in 2-L foil-covered conical flasks at 25°C, with 16-h light (cool-white fluorescent light at 70 μmol m^−2^ s^−1^) and 8-h dark cycling and constant shaking on a rotary shaker (160 rpm) (3, 25). The growth and numbers of *N. punctiforme* cells were assessed based on absorbance at 665 nm using chlorophyll (Chl) *a* (3, 31) and viability by treating cells with Trypan Blue and performing cell counts (25, 26).

### Growth conditions for *B. napus* L. (canola)

In three independent experiments, five surface-sterilized *B. napus* seeds (Pioneer Hybrid 45Y66 cultivar; donated by Elders, Geelong, Australia) were germinated and grown in CYG plant germination pouches (Mega International, Newport, MN, USA) with 20 mL of modified Hoagland and Arnon (23) medium at 22°C in a phytotron for a 2-week growth period with a 16:8-h light:dark cycle. In the co-culturing of *P. fluorescens* with *B. napus* and *N. punctiforme*, Hoagland and Arnon medium (23) was modified with the addition of 2 M KNO_3_, 1 M Ca(NO_3_)_2_4H_2_O, and 1 M NH_4_NO_3_.

### Co-cultivation of *B. napus* with *P. fluorescens* DUS1-27 and *N. punctiforme* strains

Growth pouches (CYG Seed Germination Pouch; Mega International) were set up with five seeds in each pouch as described above. In all growth assays, 20 mL of modified Hoagland and Arnon (23) medium was added to each pouch for the following treatments: control (no bacteria added, *B. napus* only); 800 μM H_2_O_2_; 150 nM HRP and *P. fluorescens* DUS1-27 (~1×10^3^ CFU mL^−1^); *P. fluorescens* DUS1-27 (~1×10^3^ CFU mL^−1^); *P. fluorescens* DUS1-27 and *N. punctiforme* WT (~1×10^3^ cells mL^−1^ final density each); *P. fluorescens* DUS1-27 and the *N. punctiforme* catalase (*Npun_R4582*^−^) knockout mutant (~1×10^3^ cells mL^−1^ final density each); and *P. fluorescens* DUS1-27 and the *N. punctiforme* catalase-overexpressing (*Npun_R4582* OE) mutant (~1×10^3^ cells mL^−1^ final density each).

### Amperometric quantification of total H_2_O_2_ levels in hydroponic growth media

Horseradish peroxidase (HRP) was immobilized on DRP-C110 screen-printed carbon electrodes (DropSens, Metrohm, Australia) following previously described methods (27, 41–43) and continuously calibrated against fresh standard H_2_O_2_ solutions. H_2_O_2_ concentrations in hydroponic media from the growth pouches for the different treatments were assessed by aspirating 1 mL of growth medium from the growth pouches into a 2-mL cuvette. An electrode (prepared as defined above) was submerged into the 2-mL cuvette containing 1 mL of medium and H_2_O_2_ levels were quantified based on the voltage through the sensor, which was measured using a Gamry Interface 1000 potentiostat (Gamry, Warminster, PA, USA) as previously described (27).

### 3,3′-Diaminobenzidine (DAB) assay for quantification of total peroxidase activity levels in hydroponic media

Peroxidase assays based on the fluorescence of DAB were modified from a previous study (4). Media (2 mL) from growth pouches were added to a 5-mL tube and spiked with 400 mM of H_2_O_2_. One hundred microliters of these samples was then mixed with 100 μL of 1 mg mL^−1^ DAB and 10 μL of 22.73 nM HRP. Samples were read at a fluorescence wavelength of 540 nm using a Polar Star Omega plate reader (BMG Labtech, Victoria, Australia) and compared against standards of known HRP concentrations ranging between 2.27 μM and 2.27×10^−6^ μM.

### Visualization of peroxidase activity in *B. napus* roots using DAB staining

Fresh root tissue from *B. napus* plants, harvested after 2 weeks of growth in hydroponic pouches, was submerged in the DAB stain (1 mg mL^−1^ of DAB in 0.1 M citrate buffer [pH 3.7]) (Sigma Aldrich, St. Louis, MO. USA) for 30 min following previous methods (4) and visualized using an inverted confocal scanning laser microscope (Leica DM IRE2Mod. no. 0871 with Leica confocal software v2.00Build) at a wavelength of 540 nm (4).

### Identification and retrieval of putative catalase gene sequences for *B. napus* and *P. fluorescens*

The complete genome sequence of *B. napus* (NCBI Reference Sequence assembly accession: GCF_000686985.1) was screened for sequences with high identity to the catalase proteins from a range of plants, including *Arabidopsis thaliana*, *Zea mays* L., and *Nicotiana tabacum*, using the Basic Local Alignment Search Tool (BLAST) (10, 18, 32, 44). Primers were designed for quantitative real-time PCR (qRT-PCR) experiments using the retrieved nucleotide sequences for the putative catalases in *B. napus: cat1* (accession number XM_013848674.1), *cat2* (accession number EU487186.1), and *cat3* (accession number JN163870.1) (Table 1).

The genome sequence for *P. fluorescens* strain F311 (taxid: 1114970, NCBI Reference Sequence assembly accession: GCF_000237065.1) was used as a template, based on it having the closest 16s rRNA gene sequence identity (>98% similarity). The NCBI-deposited *P. fluorescens* strain F311 genome was screened using BLAST for sequences with high identity to catalase proteins from a range of bacteria including *Sinorhizobium meliloti*, *Escherichia coli*, *N. punctiforme*, *P. aeruginosa*, and *P. putida* (9, 21, 22, 27, 35, 36, 46). In qRT-PCR experiments, primers were designed using the retrieved sequences for the putative catalases: *katA* (accession number WP_014340471.1), *katB* (accession number WP_014340303.1), and *katE* (accession number WP_014335811.1) (Table 1).

### Extraction and purification of RNA and synthesis of complementary DNA (cDNA)

The extraction and purification of RNA from *B. napus* samples was performed using 100 mg (wet weight) of freshly harvested plant tissue frozen in liquid nitrogen ground to a paste with a mortar and pestle with the addition of 1 μL of Protector RNase inhibitor. Subsequent isolation and purification was achieved using the QIAGEN RNeasy Plant Mini Kit, following a previously described and modified protocol (25, 27, 48).

Regarding *P. fluorescens* samples, hydroponic growth medium (20 mL) was poured from pouches after the 2-week treatment period into 50-mL plastic tubes. Cells suspended in hydroponic growth medium were pelleted out by centrifugation at 5,000×*g* at 4°C for 5 min, and 19 mL of the supernatant (hydroponic growth medium) was then aspirated. Cell pellets were resuspended in the remaining 1 mL of liquid to a cell density of ~1×10^9^ cells mL^−1^. The extraction and purification of RNA was performed using 1 mL of the 1×10^9^ cells obtained according to the manufacturer’s protocol for the QIAGEN RNeasy Mini Kit.

Following extraction, RNA from *B. napus* and *P. fluorescens* samples was further purified using the Ambion DNA-free kit according to the manufacturer’s protocol (Ambion, Waltham, MA, USA) with 1 μL of Protector RNase inhibitor added to RNA after the Ambion DNA-free treatment. Total RNA concentrations were assessed using a NanoDrop spectrophotometer (ThermoFisher Scientific, Waltham, MA, USA). cDNA synthesis was achieved using the High-capacity cDNA Reverse Transcription Kit as per the manufacturer’s protocol (ThermoFisher Scientific).

### Measurement of changes from relative mRNA levels for catalase genes using qRT-PCR

qRT-PCR was used to investigate the transcriptional responses of *cat1*, *cat2*, and *cat3* and chloroplast-localized catalase (*cat_chloroplast*) genes in *B. napus* as well as *katA*, *katE*, and *katB* in *P. fluorescens* DUS1-27. Housekeeping primers targeting *actin-2* (*act2*) in *B. napus* and the 16S rRNA gene of *P. fluorescens* DUS1-27 were also produced. Primers were designed using Primer Express (v2.0 for Windows 2000; Applied Biosystems, Foster City, CA, USA) and tested using established methods (25, 27, 48) (Table 1). Primer binding efficiencies were established using 1, 2, 4, and 8 μg mL^−1^ of template cDNA as a control and then changes in the cycle times (ΔCt) of amplification against increasing cDNA concentrations were compared.

qRT-PCR was completed according to the manufacturer’s protocol using 1×SYBR Green Master Mix (Applied Biosystems), 20 ng of the cDNA template, and 0.3 μM of forward and reverse primers. qRT-PCR analyses were conducted using the Applied Biosystems 7500 Real Time PCR system and Biosystems 7500 SDS software (Applied Biosystems). qRT-PCR stages were followed as previously described (25, 27, 48): Stage 1. 50°C for 2 min, Stage 2. 95°C for 3 min, Stage 3. 95°C for 15 s followed by 60°C for 45 s, repeated for 40 cycles, and Stage 4 (dissociation step) 95°C for 15 s, 60°C for 1 min, and 95°C for 15 s.

### Statistical analysis

Statistical analyses were based on normally distributed data produced from three independent replicate experiments of five plants each. The statistical program IBM SPSS Statistics 25 (for Windows) was used for all statistical analyses. Probability plots (P-P Plots) were produced for all data sets to test for normal distribution. Multiple comparisons of means were performed by a one-way analysis of variance (one-way ANOVA) and Tukey’s honest significant difference test for plant and bacterial growth, the quantification of peroxidase activity, H_2_O_2_ quantification assays, and qRT-PCR data sets. All statistical analyses were tested against the probability value (*P*-value) of <0.05.

## Results

### Effects of *P. fluorescens* DUS1-27 or *P. fluorescens* DUS1-27 and *N. punctiforme* on *B. napus* plant growth

**Fig. 1:**
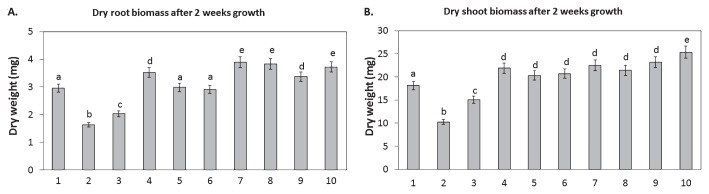
Average dry root (**A**) and shoot (**B**) biomasses of *B. napus* plants (*n*=3) after 2 weeks of growth in a hydroponic system with the following treatments: 1. control, 2. inoculated with *P. fluorescens* DUS1-27, 3. treated with 800 μM H_2_O_2_, 4. treated with 150 nM of HRP and inoculated with *P. fluorescens* DUS1-27, 5. inoculated with *N. punctiforme* WT, 6. Inoculated with *N. punctiforme NpunR4582*^−^, 7. inoculated with *N. punctiforme NpunR4582* OE, 8. co-inoculated with *P. fluorescens* DUS1-27 and *N. punctiforme* WT, 9. co-inoculated with *P. fluorescens* DUS1-27 and *N. punctiforme NpunR4582*^−^, and 10. co-inoculated with *P. fluorescens* DUS1-27 and *N. punctiforme NpunR4582* OE. Different letters on treatments indicate significant differences (*P*<0.05) between the means of treatment groups.

*B. napus* seeds inoculated with bacterial cells were grown in a hydroponic growth system for 2 weeks, and dry root and shoot biomasses were then measured (Fig. 1A and B). The inoculation of plants with *P. fluorescens* DUS1-27 significantly reduced root and shoot biomasses by 1.3 and 7.9 mg, respectively (Fig. 1A and B). The addition of 800 μM of H_2_O_2_ significantly reduced root and shoot biomasses by 0.9 and 3 mg, respectively, from those of control plants; however, these root and shoot biomasses were still significantly higher than those of inoculated plants (~0.4 mg for roots and ~5 mg for shoots). The addition of 150 nM HRP to *B. napus* inoculated with *P. fluorescens* DUS1-27 significantly increased plant root and shoot biomasses by 0.6 and 3.8 mg, respectively, over those of control plants (Fig. 1A and B).

The inoculation of *B. napus* with *N. punctiforme* WT or *N. punctiforme Npun_R4582*^−^ significantly increased the shoot biomass over that of the control, by approximately 2 mg, after 2 weeks, and this biomass was similar to that of plants treated with 150 nM HRP (Fig. 1B). The inoculation of *B. napus* with *N. punctiforme Npun_R4582* OE significantly increased root and shoot biomasses by ~1 and ~4 mg, respectively, with root biomasses being significantly higher (~0.3 mg) for these plants than for HRP-treated plants (Fig. 1A and B).

The co-inoculation of *B. napus* with *P. fluorescens* DUS1-27 and *N. punctiforme* WT, *N. punctiforme Npun_R4582*^−^, or *N. punctiforme Npun_R4582* OE significantly increased root dry biomasses by ~1, ~0.5, and ~0.8 mg, respectively, over that of the control (Fig. 1A). The co-inoculation of *B. napus* with *P. fluorescens* and *N. punctiforme* WT, *N. punctiforme Npun_R4582*^−^, or *N. punctiforme Npun_R4582* OE significantly increased dry shoot biomasses by ~3.3, ~5, and ~7 mg, respectively, over that of the control (Fig. 1B). Root biomass was the highest for plants inoculated with *N. punctiforme Npun_R4582* OE or co-inoculated with *P. fluorescens* DUS1-27 and *N. punctiforme* WT; root biomass was significantly higher than that of control plants or plants treated with 150 nM HRP (Fig. 1A). The co-inoculation with *P. fluorescens* DUS1-27 and *N. punctiforme Npun_R4582* OE resulted in the highest biomass yielded (Fig. 1B).

### Quantification of H_2_O_2_ levels in hydroponic growth medium from *B. napus* inoculated with *P. fluorescens* DUS1-27 or co-inoculated with *P. fluorescens* DUS1-27 and *N. punctiforme*

**Fig. 2:**
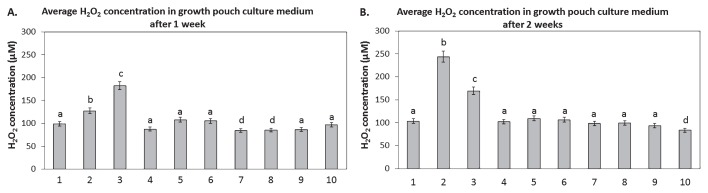
Amperometric quantification of hydrogen peroxide (H_2_O_2_) concentrations in hydroponic growth media (from three independent samples for each treatment, *n*=3) performed after 1 week (**A**) and 2 weeks (**B**) of growth for the following treatments: 1. control, 2. inoculated with *P. fluorescens* DUS1-27, 3. treated with 800 μM H_2_O_2_, 4. treated with 150 nM of HRP and inoculated with *P. fluorescens* DUS1-27, 5. inoculated with *N. punctiforme* WT, 6. inoculated with *N. punctiforme NpunR4582*^−^, 7. inoculated with *N. punctiforme NpunR4582* OE, 8. co-inoculated with *P. fluorescens* DUS1-27 and *N. punctiforme* WT, 9. co-inoculated with *P. fluorescens* DUS1-27 and *N. punctiforme NpunR4582*^−^, and 10. co-inoculated with *P. fluorescens* DUS1-27 and *N. punctiforme NpunR4582* OE. Different letters on treatments indicate significant differences (*P*<0.05) between the means of treatment groups.

The growth media of plants inoculated with *P. fluorescens* DUS1-27 had significantly higher levels of H_2_O_2_ after 1 and 2 weeks of growth than that of uninoculated (control) plants (Fig. 2A and B). H_2_O_2_ levels after 1 week were ~30 μM higher in growth media from plants inoculated with *P. fluorescens* DUS1-27 and were ~140 μM higher after 2 weeks (Fig. 2A and B). The elevated H_2_O_2_ levels detected in growth media from plants inoculated with *P. fluorescens* DUS1-27 correlated with the significant reductions observed in root and shoot biomasses after 2 weeks (Fig. 1A and B). After 1 week, growth media treated with 800 μM of H_2_O_2_ had significantly higher H_2_O_2_ levels than those from all other treatments, whereas growth media from plants inoculated with *P. fluorescens* DUS1-27 had significantly higher H_2_O_2_ levels after 2 weeks than those from all other treatments (Fig. 2A and B).

Overall, growth media from plants treated with 150 nM HRP and inoculated with *P. fluorescens* DUS1-27, inoculated with *N. punctiforme* strains only, or co-inoculated with *N. punctiforme* strains and *P. fluorescens* DUS1-27 had similar H_2_O_2_ levels to that from the uninoculated control (Fig. 2A and B). H_2_O_2_ levels in growth media from plants inoculated with *N. punctiforme Npun_R4582* OE or co-inoculated with *P. fluorescens* and *N. punctiforme* WT were significantly lower (49.7% decrease in H_2_O_2_ concentrations) after 1 week than in those from all other treatments (Fig. 2A). After 2 weeks, growth media from plants co-inoculated with *P. fluorescens* DUS1-27 and *N. punctiforme Npun_R4582* OE had significantly lower H_2_O_2_ levels (at least 20 μM lower) than those from all other treatments (Fig. 2B). These results demonstrate that the addition of *N. punctiforme*, particularly the *Npun_R4582* OE strain, reduced H_2_O_2_ levels in growth media, thereby reducing growth-limiting oxidative stress in plants (as shown in Fig. 1A and B).

### Quantification of total peroxidase activity levels in growth media from *B. napus* inoculated with *P. fluorescens* or co-inoculated with *P. fluorescens* DUS1-27 and *N. punctiforme*

**Fig. 3:**
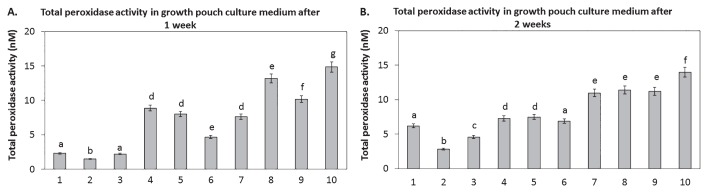
Quantification of total peroxidase activity levels in hydroponic growth media (from three independent samples for each treatment, *n*=3) after 1 week (**A**) and 2 weeks (**B**) of growth using 3,3′-diaminobenzidine (DAB) measured at a wavelength of 540 nm for the following treatments: 1. control, 2. inoculated with *P. fluorescens* DUS1-27, 3. treated with 800 μM H_2_O_2_, 4. treated with 150 nM of HRP and inoculated with *P. fluorescens* DUS1-27, 5. inoculated with *N. punctiforme* WT, 6. inoculated with *N. punctiforme NpunR4582^−^*, 7. inoculated with *N. punctiforme NpunR4582* OE, 8. co-inoculated with *P. fluorescens* DUS1-27 and *N. punctiforme* WT, 9. co-inoculated with *P. fluorescens* DUS1-27 and *N. punctiforme NpunR4582*^−^, and 10. co-inoculated with *P. fluorescens* DUS1-27 and *N. punctiforme NpunR4582* OE. Different letters on treatments indicate significant differences (*P*<0.05) between the means of treatment groups.

Total peroxidase activity levels in growth media were measured to establish whether *N. punctiforme* was capable of reducing the plant growth-inhibiting levels of H_2_O_2_ (Fig. 3). After 1 week, growth media from plants inoculated with *P. fluorescens* DUS1-27 had significantly lower peroxidase activity levels than the uninoculated control and those from all other treatments (Fig. 3A). The addition of 150 nM HRP to plants inoculated with *P. fluorescens* DUS1-27, with *N. punctiforme* strains, or co-inoculated with the *P. fluorescens* DUS1-27 and *N. punctiforme* strains resulted in significantly higher total peroxidase activity levels of more than 50% from those in media from uninoculated control plants and plants inoculated with *P. fluorescens* DUS1-27 only (Fig. 3A). The addition of 800 μM H_2_O_2_ resulted in significantly lower total peroxidase activity levels than those of the control after 2 weeks only, with peroxidase levels still being higher for this treatment than in growth media from plants inoculated with *P. fluorescens* DUS1-27 (Fig. 3B). After 2 weeks, total peroxidase levels in growth media from *B. napus* inoculated with *P. fluorescens* DUS1-27 were significantly lower at 50% of those in growth media from the control (Fig. 3B). Plants co-inoculated with *N. punctiforme* strains plus *P. fluorescens* DUS1-27 had significantly higher total peroxidase activity levels than uninoculated (control) plants and plants inoculated with *P. fluorescens* DUS1-27 only (Fig. 3B). Among the three *N. punctiforme* strains tested (WT, *Npun_R4582*^−^, and *Npun_R4582* OE), the *Npun_R4582* OE strain increased total peroxidase activity levels the most, by more than 50% compared to growth media from control plants and plants treated with 150 nM HRP and inoculated with *P. fluorescens* (Fig. 3B). Total peroxidase activity levels in growth media from plants co-inoculated with *P. fluorescens* DUS1-27 and *N. punctiforme Npun_R4582* OE were significantly higher than in those from all other treatments after 2 weeks (Fig. 3A and B).

### Visualization of peroxidase activity in roots from *B. napus* plants inoculated with *P. fluorescens* or co-inoculated with *P. fluorescens* DUS1-27 and *N. punctiforme*

**Fig. 4:**
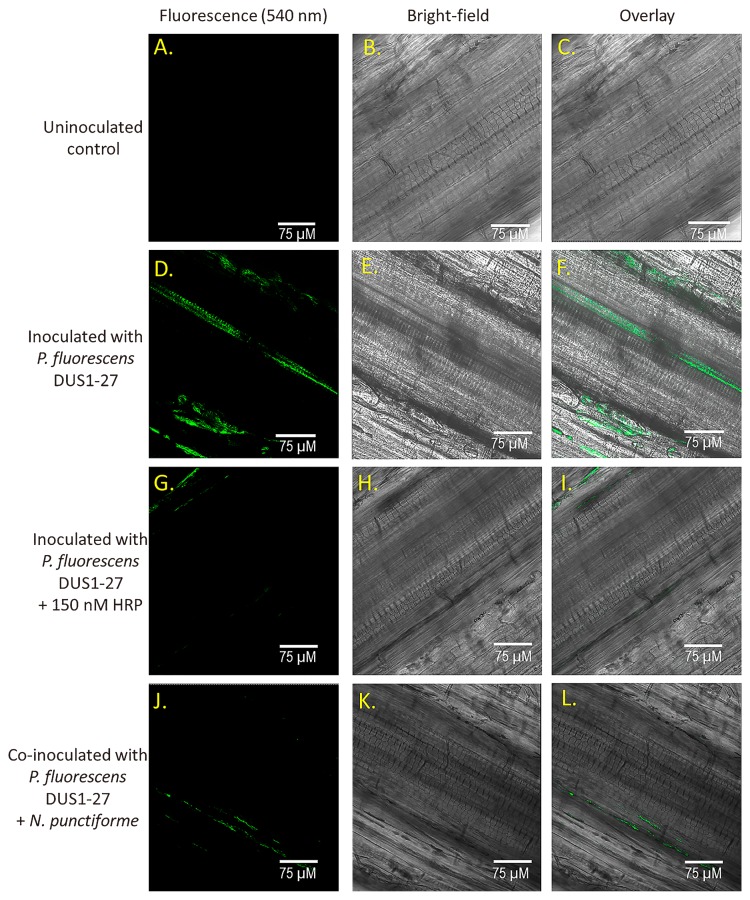
Visualization of total peroxidase activity levels in roots (stained with DAB and visualized at 540 nm, in green) of *B. napus* plants grown in a hydroponic system for 2 weeks. Bright field images of the plant root cells and overlay images of fluorescence and bright-field images were also recorded. Control *B. napus* plant roots (uninoculated and no added HRP) (**A, B, and C**), roots from *B. napus* inoculated with *P. fluorescens* DUS1-27 (**D, E, and F**), *B. napus* roots from plants inoculated with *P. fluorescens* DUS1-27 and treated with peroxidase (150 nM HRP) (**G, H, and I**), and roots from *B. napus* plants co-inoculated with both *P. fluorescens* DUS1-27 and *N. punctiforme* (**J, K, and L**).

Plants inoculated with *P. fluorescens* DUS1-27 had higher levels of fluorescence than uninoculated (control) plants after 2 weeks of growth (Fig. 4A, B, C, D, E, and F). Plants grown in media spiked with HRP (150 nM) and inoculated with *P. fluorescens* DUS1-27 or plants co-inoculated with *P. fluorescens* DUS1-27 and *N. punctiforme* had lower peroxidase activity levels than uninoculated (control) plants (Fig. 4A, B, C, G, H, I, J, K, and L). Roots from plants co-inoculated with *P. fluorescens* DUS1-27 and *N. punctiforme* or treated with HRP (150 nM) and *P. fluorescens* DUS1-27 had markedly lower peroxidase activity levels (Fig. 4G, H, I, J, K, and L) than roots from plants inoculated with only *P. fluorescens* DUS1-27 (Fig. 4D, E, and F).

### Catalase gene expression in roots and shoots of *B. napus* inoculated with *P. fluorescens* DUS1-27 or co-inoculated with *P. fluorescens* DUS1-27 and *N. punctiforme*

**Fig. 5:**
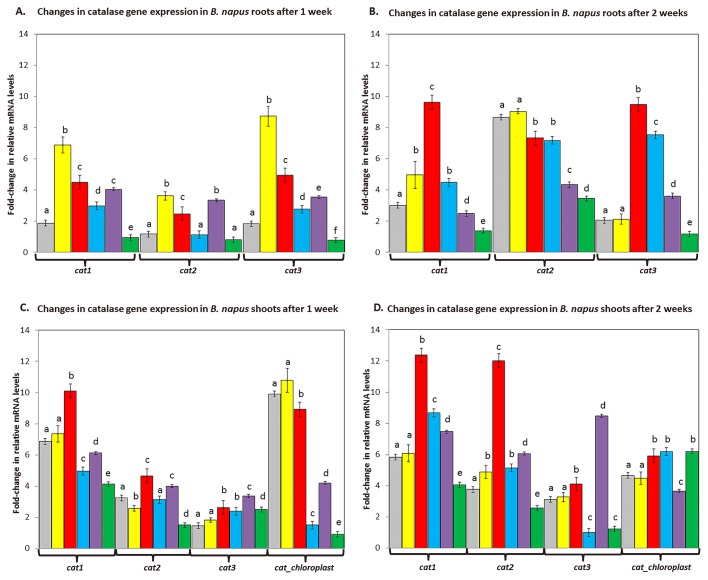
Changes in relative mRNA levels for catalase genes *cat1*, *cat2*, and *cat3* and a chloroplast-localized catalase (*cat*_chloroplast) in *B. napus* roots and shoots from three independent samples for each treatment (*n*=3) were measured after 1 week (**A**) and 2 weeks (**B**) of growth with the following treatments: uninoculated *B. napus* control plants (grey), *B. napus* treated with 800 μM H_2_O_2_ (yellow), *B. napus* inoculated with *P. fluorescens* DUS1-27 (red), *B. napus* co-inoculated with *P. fluorescens* DUS1-27 and *N. punctiforme* WT (blue), *B. napus* co-inoculated with *P. fluorescens* DUS1-27 and *N. punctiforme NpunR4582^−^* (purple), and *B. napus* co-inoculated with *P. fluorescens DUS1-27* and *N. punctiforme NpunR4582* OE (green). Different letters on treatments indicate significant differences (*P*<0.05) between the means of treatment groups.

Changes in relative mRNA levels for the catalase genes *cat1*, *cat2*, and *cat3* in *B. napus* treated with 800 μM H_2_O_2_ were quantified and significant increases were observed in their expression levels, particularly in roots from plants treated for 1 week, with at least a 3-fold increase being noted in the expression levels of all genes from those in uninoculated control plants (Fig. 5A). After 2 weeks, the exogenous H_2_O_2_ treatment only significantly altered *cat1* relative mRNA levels, which were 0.6-fold higher than the control level (Fig. 5B). The addition of 800 μM of H_2_O_2_ did not alter catalase gene expression in shoots from that in the control (Fig. 5C and D).

The effects of the inoculation with *P. fluorescens* DUS1-27 on *B. napus* relative mRNA levels for catalase genes (*cat1*, *cat2*, and *cat3*) were investigated. After 1 week, the relative mRNA levels of *cat1*, *cat2*, and *cat3* in roots were significantly higher in plants inoculated with *P. fluorescens* DUS1-27 (~2-fold higher) than in uninoculated (control) plants (Fig. 5A). After 2 weeks, the addition of *P. fluorescens* DUS1-27 significantly increased the relative mRNA levels of *cat1* and *cat3* further, by approximately 3-fold, over control levels (Fig. 5B). After 2 weeks of growth, *B. napus cat2* relative mRNA levels were significantly lower in *P. fluorescens* DUS1-27-inoculated and co-inoculated plant roots (Fig. 5B).

The effects of co-inoculating with *P. fluorescens* DUS1-27 and *N. punctiforme* on *B. napus* relative mRNA levels for catalase genes (*cat1*, *cat2* and *cat3*) were investigated, with changes in overall expression profiles being observed. The co-inoculation of *B. napus* with *P. fluorescens* DUS1-27 and *N. punctiforme* WT significantly increased *cat1* and *cat3* relative mRNA levels, while *cat2* levels remained similar to control levels (Fig. 5A). The addition of *N. punctiforme* WT significantly reduced *B. napus* catalase gene expression levels after 1 week from those in H_2_O_2_-treated and *P. fluorescens* DUS1-27-inoculated plants (Fig. 5A). After 2 weeks, all co-inoculation treatments (except for *cat2* in roots from plants co-inoculated with *N. punctiforme* WT) resulted in lower catalase relative mRNA levels in roots than those in *P. fluorescens* DUS1-27-inoculated plant roots (Fig. 5B).

The co-inoculation of plants with *P. fluorescens* DUS1-27 and *N. punctiforme Npun_R4582*^−^ resulted in significantly higher relative mRNA levels, by up to 2-fold, in roots after 1 week than in uninoculated (control) plants (Fig. 5A). After 2 weeks, relative mRNA levels for all root catalases in *B. napus* co-inoculated with *N. punctiforme Npun_R4582*^−^ were reduced by ~2-fold (Fig. 5B). The co-inoculation of *B. napus* with *P. fluorescens* DUS1-27 and *N. punctiforme Npun_R4582* OE most effectively reduced relative mRNA levels in roots over 2 weeks, with all catalase gene expression levels being significantly lower than those with the control and all other treatments (Fig. 5A and B). In *B. napus* shoots, the relative mRNA levels of *cat1*, *cat2*, and *cat3* after 1 and 2 weeks of the inoculation with *P. fluorescens* DUS1-27 were significantly higher, by up to 2-fold, than those in uninoculated control plants (Fig. 5C). The relative mRNA levels of chloroplast-associated catalase *cat*_chloroplast were significantly lower for all inoculation strategies after 1 week than in control plants (Fig. 5C). After 1 week of treatment, the relative mRNA levels of *cat1*, *cat2* and *cat*_chloroplast were all significantly lower in the shoots of plants co-inoculated with *P. fluorescens* DUS1-27 and *N. punctiforme Npun_R4582* OE than in uninoculated control plants (Fig. 5C). Apart from *cat3*, the co-inoculation reduced the expression levels of all catalase genes to lower than those in plants inoculated with *P. fluorescens* DUS1-27 only (Fig. 5C). After 2 weeks, the relative mRNA levels of *cat1*, *cat2*, and *cat3* were all significantly lower in shoots from plants co-inoculated with *P. fluorescens* DUS1-27 and *N. punctiforme Npun_R4582* OE than in uninoculated control plants (Fig. 5D). Besides plants co-inoculated with *P. fluorescens* DUS1-27 and *N. punctiforme Npun_R4582*^−^, *cat*_chloroplast relative mRNA levels after 2 weeks were significantly higher in inoculated and co-inoculated plants than in control plants (Fig. 5D).

### Changes in the expression of catalase genes in *P. fluorescens* DUS1-27

**Fig. 6:**
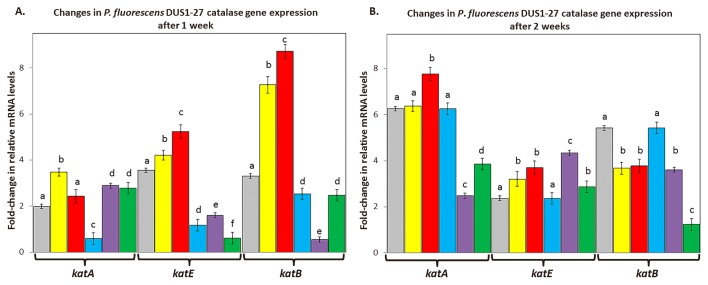
Changes in relative mRNA levels for catalase genes *katA*, *katE*, and *katB* in *P. fluorescens* DUS1-27 associated with *B. napus* plants roots were measured after 1 week (**A**) and 2 weeks (**B**) of growth in three independent samples (*n*=3) for each of the following treatments: *Pseudomonas fluorescens* DUS1-27 cultured without *B. napus* (grey), cells treated with 800 μM H_2_O_2_ (yellow), cells inoculated with *B. napus* (red), cells from *B. napus* co-inoculated with *P. fluorescens* DUS1-27 and *N. punctiforme* WT (blue), cells from *B. napus* co-inoculated with *P. fluorescens* DUS1-27 and *N. punctiforme NpunR4582*^−^ (purple), and cells from *B. napus* co-inoculated with *P. fluorescens* DUS1-27 and *N. punctiforme NpunR4582* OE (green). Different letters on treatments indicate significant differences (*P*<0.05) between the means of treatment groups.

*P. fluorescens* DUS1-27 *katA*, *katE*, and *katB* gene expression levels were significantly higher after 1 week when growth media contained 800 μM of added H_2_O_2_ (Fig. 6A). In the presence of *B. napus*, *P. fluorescens* DUS1-27 *katE* and *katB* gene expression levels were significantly higher than both control levels (cells in hydroponic medium without *B. napus* plants or *N. punctiforme* cells) and cells treated with 800 μM of added H_2_O_2_ (Fig. 6A). After 2 weeks, *katA* and *katE* relative mRNA levels in cells grown with *B. napus* were significantly higher than control levels, whereas *katB* relative mRNA levels were significantly lower (Fig. 6B). In cells treated with 800 μM of added H_2_O_2,_ *katE* relative mRNA levels were significantly higher than control levels, whereas *katB* expression levels were significantly lower, with both genes showing similar expression levels to cells inoculated with *B. napus* (Fig. 6B). The relative mRNA levels of *katE* and *katB* were significantly lower with the co-inoculated treatments than with the control treatment (*P. fluorescens* only) and *P. fluorescens* grown with *B. napus* (Fig. 6A). Catalase relative mRNA levels in cells co-inoculated with *N. punctiforme Npun_R4582* OE were significantly lower, by up to 2-fold, than in cells grown in conjunction with *B. napus* (Fig. 6B).

## Discussion

*Pseudomonas* species, including *P. brassicacearum*, *P. asplenii*, and *P. putida*, exert beneficial effects on the growth of plants, including *B. napus* (7, 37, 39, 49). In the present study, using a hydroponic growth system, the inoculation of *B. napus* with *P. fluorescens* DUS1-27 exerted deleterious effects on plant growth, even though this isolate has previously been shown to promote plant growth in soil-based systems. The examination of plant germination and seedling physiology showed wilted chlorotic cotyledons after 1 to 2 weeks of growth, despite an identical nutrient status to uninoculated control plants. Based on these findings, oxidative stress was proposed as the factor responsible for reducing plant growth (20, 53). The detection of significantly elevated levels of H_2_O_2_ in growth media supported this.

The elevated total peroxidase activity levels observed in roots suggested that plants were responding to oxidative stress by increasing catalase/peroxidase production. Plants produce ROS during normal metabolic processes, predominantly photosynthesis and photorespiration, which are then broken down by antioxidant enzymes (38, 47). Low levels of H_2_O_2_ play important roles in plant cell development by activating signaling pathways and transcription factors, which, in turn, regulate gene expression and cell cycle processes (6, 38). The regulation of H_2_O_2_ production and environmental levels in close proximity to plants (within the plant rhizosphere) plays an important role in regulating signaling pathways and transcription factors, and also forms part of the plant pathogen defense mechanism (6, 29, 34, 38). The increased H_2_O_2_ levels measured in the hydroponic medium of *B. napus* inoculated with *P. fluorescens* DUS1-27 suggests that bacteria were exerting pathogenic or MAMP-like effects on plants, triggering plant defense mechanisms. To mitigate increased ROS levels, plants increased the production of peroxidase enzymes, as demonstrated by our qRT-PCR data (6, 29, 30, 34, 38). Therefore, catalase gene transcription levels were measured using qRT-PCR in *B. napus* plants exposed to *P. fluorescens* DUS1-27. In *A. thaliana*, a small gene family encodes three catalase proteins, Cat1 (Class II), Cat2 (Class I), and Cat3 (Class III) (14). These extensively examined systems of *A. thaliana* are representative of the catalase systems in most plants, including those of *B. napus* (14). In *B. napus* inoculated with *P. fluorescens* DUS1-27, relative mRNA levels for catalase genes (*cat1*, *cat2*, and *cat3*) generally increased in roots and shoots. This corresponds to the increased total peroxidase activity levels observed in roots, suggesting that plants were attempting to regulate the increased cellular levels of H_2_O_2_ caused by the exposure to *P. fluorescens* DUS1-27.

The present results provide unique insights into the mechanisms by which bacteria that exhibit PGPB effects under soil-based growth systems trigger MAMP-like responses, leading to plant-growth inhibiting oxidative stress in confined systems, such as those of our hydroponic system. The hydroponic growth system was initially employed in this study to reduce the variability associated with plant growth in soil-based systems. This allows absolute control over growth conditions and a greater range of molecular and biochemical analyses (50, 52, 53). However, as substantiated by the present results and previous findings (50, 52, 53), the hydroponic system altered the dynamics of the plant-microbe interaction, which was ultimately deleterious for plants and not representative of the soil-based system.

The co-inoculation of *B. napus* with *P. fluorescens* DUS1-27 and three different strains of *N. punctiforme* that exhibit various catalase/peroxidase activity levels was investigated to clarify whether cyanobacteria offset the oxidative stress caused by the *P. fluorescens* strain. Plants inoculated with *N. punctiforme* strains or co-inoculated with *N. punctiforme* strains plus *P. fluorescens* DUS1-27 showed significantly more growth and higher total peroxidase activity levels than uninoculated (control) plants or plants inoculated with *P. fluorescens* DUS1-27 only. This result demonstrates that *N. punctiforme* has the capacity to enhance plant growth by reducing oxidative stress caused by increased H_2_O_2_ levels, which were likely produced as a defense response by plants towards *P. fluorescens* DUS1-27.

*B. napus* co-inoculated with *P. fluorescens* and *N. punctiforme* strains had lower relative mRNA levels of the catalase genes *cat1*, *cat2*, and *cat3* than plants inoculated with *P. fluorescens* only. This result also indicates that the addition of high catalase-producing *N. punctiforme* reduced H_2_O_2_ in this system, enabling normal growth in the presence of the otherwise growth-inhibiting *P. fluorescens*. It is also clear that while the knockout of one of the *N. punctiforme* catalase genes (*Npun_R4582*^−^) reduced overall peroxidase activity, sufficient peroxidase activity was still available. This is most likely via a compensatory response by other catalase genes (at least seven) in the *N. punctiforme* genome (27). The cell number of *P. fluorescens* DUS1-27 (~1.3×10^6^) was not significantly altered by the co-inoculations with any of the *N. punctiforme* strains, and no changes were observed in the number of *N. punctiforme* cells (~3×10^5^) co-inoculated with *P. fluorescens* DUS1-27 (Table 2). This result suggests that *P. fluorescens* did not induce pathogenesis towards *N. punctiforme*, and, likewise, *N. punctiforme* did not restore canola growth via toxicity towards *P. fluorescens*.

The addition of the *N. punctiforme Npun_R4582* OE strain was the most effective at offsetting the negative interaction between *B. napus* and *P. fluorescens* DUS1-27 in the hydroponic system. The co-inoculation with the *N. punctiforme Npun_R4582* OE strain resulted in increased peroxidase activity levels in the growth system, which, in turn, reduced H_2_O_2_ levels in growth media, decreased peroxidase activity levels in plant roots, and alleviated the burden on the plant and *P. fluorescens* DUS1-27 molecular response mechanisms, resulting in plant growth being among the greatest observed across all treatments. In all cases, the *N. punctiforme* inoculation resulted in enhanced root and shoot biomasses after 2 weeks. Plant catalase production and, ultimately, plant growth were clearly influenced by the oxidative stress associated with the accumulation of H_2_O_2_ in the hydroponic system when plants were challenged with *P. fluorescens* DUS1-27. Based on the present results showing that *N. punctiforme* reduced the growth-inhibiting levels of H_2_O_2_ in our growth system, and other previous *in vivo* findings on cyanobacterial catalase and peroxidase activities (51), we propose that cyanobacteria, such as *N. punctiforme*, contribute to enhancing crop growth through their production of high levels of antioxidants, including catalases and peroxidases. Moreover, our results demonstrated for the first time that the restoration of plant growth under ROS stress may be achieved by co-inoculating with *N. punctiforme*, most likely due to its endogenous peroxidase activity.

## Figures and Tables

**Table 1:** Primers for the amplification of catalase genes in *B. napus* and *P. fluorescens*. Forward and reverse primers were denoted by ‘F’ and ‘R’ respectively.

Primer name	Primer sequence (5′ to 3′ orientation)
*P. fluorescens* 16S F	ACGCCGTAAACGATGTCAACTA
*P. fluorescens* 16S R	TTAACCTTGCGGCCGTACTC
*P. fluorescens* Catalase *katA* F	CTTTGGCAGCCACACCTACA
*P. fluorescens* Catalase *katA* R	CCGGCGCCAGGTTCTT
*P. fluorescens katE* F	CGAGGAAGACGAGCACAACTTT
*P. fluorescens katE* R	GCGGTTCAGCACCATCTTG
*P. fluorescens katB* F	CGCTCCTTCAGCAAGAAGGA
*P. fluorescens katB* R	GTAAAGGAACGACAGCATGATGTG
*B. napus* Actin 2 F	AGAGCGGGAAATTGTAAGAGACAT
*B. napus* Actin 2 R	TCTCGATGGAGGAGCTGGTT
*B. napus cat1* F	CCCAGAGGTCCTATCCTTCTTGA
*B. napus cat1* R	GCTCCTCTTGCGTGAACCA
*B. napus cat2* F	TCCAAAGTGTGCTCACCACAA
*B. napus cat2* R	GAACCGGGTCATACCTCGAA
*B. napus cat3* F	GGGAACTTTGATCTCGTTGGAA
*B. napus cat3* R	GTTTTCGGGTTCGGCTTCA
*B. napus* CAT_Chloroplast F	CCCTCCCATCACACATGAAAT
*B. napus* CAT_Chloroplast R	CAGACGGCTTGCCAGCT

**Table 2:** Number of *P. fluorescens* and *N. punctiforme* colony-forming units mL^−1^ (CFU mL^−1^) in hydroponic growth medium after 2 weeks of growth co-inoculated with *B. napus*

Inoculant	CFU mL^−1^ of *P. fluorescens*	CFU mL^−1^ of *N. punctiforme*
*P. fluorescens*	1.35±0.32×10^6^	—
*P. fluorescens*+*N. punctiforme* WT	1.34±0.41×10^6^	3.12±0.28×10^5^
*P. fluorescens*+*N. punctiforme* Npun_R4582^−^	1.37±0.25×10^6^	2.94±0.31×10^5^
*P. fluorescens*+*N. punctiforme* Npun_R4582 OE	1.36±0.35×10^6^	3.03±0.29×10^5^

CFU mL^−1^ of *P. fluorescens*, *N. punctiforme* WT, *N. punctiforme* catalase knockout (*Npun_R4582*^−^), and *N. punctiforme* catalase-overexpressing (*Npun_R4582* OE) plants after a co-inoculation with *B. napus* for 2 weeks in hydroponic growth media. CFU mL^−1^ of *P. fluorescens* assessed using Miles and Misra plate counts. CFU mL^−1^ of *N. punctiforme* based on chlorophyll a. No significant differences (*P*<0.05) were observed in CFU mL^−1^ between treatments.
